# Fertility Outcome after Treatment of Unruptured Ectopic Pregnancy with Two Different Methotrexate Protocols

**Published:** 2012-12-17

**Authors:** Afsar Tabatabaii Bafghi, Fatemah Zaretezerjani, Leila Sekhavat, Raziah Dehghani Firouzabadi, Zeynab Ramazankhani

**Affiliations:** Department of Obstetrics and Gynecology, Shahid Sadoughi Hospital, Shahid Sadoughi University of Medical Sciences and Health Services, Yazd, Iran

**Keywords:** Ectopic Pregnancy, Methotrexate, Pragnacy Outcome, Complication

## Abstract

**Background:**

The purpose of this study was to compare the success rates of 70 patients from the same database, each with an ectopic pregnancy (EP) that was treated with either the single- or multi-dose methotrexate (MTX) protocols for unruptured EPs.

**Materials and Methods:**

This study was a blinded, randomized clinical trial. Treatment protocols were either single- (50 mg/m^2^) dose MTX or multi-dose (1 mg/kg MTX + 0.1 mg/kg folinic acid). There were 35 cases in each group. The outcome was measured by adverse events, resolution of pregnancy without surgical treatment, success rate of MTX treatment, and fertility outcome in each group.

**Results:**

With the single-dose protocol, response to treatment was considered successful in 29 (82.9%) patients; in the multi-dose protocol 31 (88.6%) responded to treatment. The difference between success rates in the groups was not statistically significant (p=0.587). In the singledose group, 2 (5.7%) patients and in the multi-dose group, 6 (17.2%) patients had complications (p=0.28). Of the 14 patients in the single-dose group. Clinical pregnancy occurred in 9 (75%) whereas clinical pregnancy occurred in 3 (25%) patients from the multi-dose group. Infertility was seen in 4 (33.3%) patients in the single-dose group and in 8 (66.7%) in the multi-dose group.

**Conclusion:**

We believe that the single-dose MTX protocol could be as successful as multi-dose MTX for the treatment of EP. It is effective, cost-effective, and associated with better fertility outcomes than the multi-dose MTX protocol (Registration Number: IRCT201112178435N1).

## Introduction

In modern obstetrics, ectopic pregnancy (EP) is a life- and fertility-threatening condition, and it remains one of the leading causes of maternal morbidity and mortality. In industrialized countries, EP is a well-documented obstetric disorder, however in developing countries its data are relatively rare and unreliable (1 ).

Approximately 90% of EPs are located in the one or both fallopian tubes, and 80% of those are located in the ampullary segment of the tubes (2 ). EP accounts for 73% of first trimester pregnancy mortalities (3 ).

However, improved modalities of early diagnosis and treatment have decreased both pregnancyrelated mortality and morbidity (4 ). Currently, the increased availability of β-human chorionic gonadotropin (β-hCG) and transvaginal ultrasound have increased the likelihood of early detection and intervention prior to tubal rupture. The use of methotrexate (MTX) as a treatment for EP has been first reported by Tanaka et al. (5 ).

Management of an unruptured EP and a single dose MTX regime for treatment was initially described by Stovall et al. (6 ). It has become a great alternative to laparoscopic surgical intervention in non-disturbed tubal EPs (7 ). MTX belongs to a class of drugs known as folic acid antagonists, which are commonly used in medical treatment of EP. The half-life of MTX is 8-15 hours for doses over 30 mg/m^2^ (8 ). Rapidly dividing cells, such as trophoblasts, are the most sensitive to MTX therapy (9 , 10 ). Some studies have reported results comparable to surgery for the treatment of EPs (11 ). EP treatment with MTX requires a followup. The success rates of medical treatment of EP vary from 75% to 95% (12 ). MTX treatment is preferred for patients with hemodynamic stability, no fetal cardiac activity at ultrasonography, pretreatment β-hCG levels less than 10000 IU/L, and normal hepatic and renal function tests (13 ).

Medical management of an unruptured EP with MTX is common and cost effective (12 ). Systemic MTX has been administered in single- and multiple- dose protocols, the previous regimen has been supported by some to improve patient compliance and reduce the side effects of treatment (14 ).

On the other hand, a meta-analysis demonstrated that multi-dose MTX was significantly better than single-dose MTX (12 ). A non-randomized prospective study that compared the two medical regimens conducted by Lipscomb et al. (15 ) showed no significant difference in failure rate between single- and multiple-dose protocols.

The single-dose regimen involves a one time administration of 50 mg/m^2^ MTX. β-hCG values are then observed on days 4 and 7. If a 15% hCG level reduction does not occur, a second dose is required (6 , 16 ). This protocol has been developed in an effort to reduce the incidence of side effects from multiple doses of MTX, eliminate the need for leucovorin rescue, and to increase the convenience of administration. Success rates for each protocol are based on extended case studies and vary among different studies, although a few studies have demonstrated comparable success rates between the two protocols (15 , 17 ).

The goal of this study was to compare the safety and success rates of single- and multiple-dose MTX protocols for the treatment of unruptured tubal EP.

## Materials and Methods

This was a blinded, randomized clinical trial study performed from 2009-2010 at Shahid Sadoughi Hospital, Yazd, Iran. The hospital is a tertiary regional and teaching hospital. The study was approved by the Committee for Ethics of Shahid Sadughi University of Medical Sciences. Institutional Review Board approval was obtained before commencing the trial as well. All patients and their husbands signed written informed consent.

### Patient selection

Diagnosis was confirmed by laparoscopic surgery and vaginal sonography. Inclusion criteria for patient selection for medical treatment were: absence of bleeding in evidences of laparoscopic surgery and vaginal sonography, stable hemodynamic state, tubal mass (estimated as the biggest diameter of entire tube with gestation seen by vaginal ultrasonography) ≤4 cm in diameter, absence of fetal heart beat, β-hCG less than 15000 mIU/mL, and leaning of patient to the next pregnancies.

To diagnose EP, patients with positive β-hCG were followed until an intrauterine pregnancy was documented. Serum β-hCG concentrations were measured at the Central Chemical Laboratory of our hospital. The assay was calibrated using the World Health Organization (WHO) Third International Standard (code 75/537) (formerly designated the WHO First International Reference Preparation). In patients who had records of β-hCG levels from other laboratories, we measured their levels again at our hospital at the beginning of treatment, and these new records were used for analyses.

An EP was diagnosed if β-hCG levels were ≥1800 mlU/mL and no viable intrauterine pregnancy was evident. Suspected EP with β-hCG levels <1800 mIU/mL was followed according to the algorithm of Stovall and Ling (14 ):

- a ≥50% increase in β-hCG over 48 hours was considered a normal intrauterine pregnancy.
- Declining β-hCG levels over 48 hours were followed by additional serial β-hCG samples and clinical status.
  These cases were considered spontaneous abortions or EP in resorption. Cases judged by the treating clinician to be an EP in resorption were not suitable to enter the study.
- Plateauing levels or a <50% increase in β-hCG over 48 hours were diagnosed as EPs. These criteria Plateauing levels or a <50% increase in β-hCG over 48 hours were diagnosed as EPs. These criteria

### Randomization

In this study, we used a computer generated block randomization method with sealed envelopes (18 ). Therefore, according to a computerized random table the patients were assigned to either singleor multiple-doses of MTX (Methotrexate, Ebewe, Unterach or Vnterach, Austria) by intramuscular injection. The numbers were kept in sealed envelopes and only opened once the decision to progress to treatment was made. The envelopes were stored and opened by an independent coordinator in an office away from the treatment center.

### Treatment protocol

#### Single-dose regimen

In the single dose regimen, 50 mg/m^2^ intramuscular MTX (Ebetrex 50 mg/ml, Ebewe Pharma Ges.m.b.h Ntg.KG, A-4866 Unterach or Vnterach, Austria) was administered on day 1 and β-hCG levels were measured on days 4 and 7. If the hCG level did not decrease by 15% between days 4 and 7, a second dose of MTX was injected on day 7. If a more than 15% decline was achieved between days 4 and 7, the β-hCG level was measured weekly until a normal level of 10 mIU/mL was obtained.

#### Multi-dose regimen

Intramuscular MTX (1 mg/kg/day) was administered on days 1, 3, 5, and 7 and citrovorum factor (0.1 mg/ kg/day) was administered on days 2, 4, 6, and 8 or after a decline in the β-hCG level on two consecutive days.

#### Outcome measures

The main outcome was a comparison of the success rates between single- and multiple-dose protocols. Success rate was defined as the percent of patients with a positive response to therapy. In the single-dose group, positive response was defined as confirmation of a 15% drop in serum β-hCG levels after one week of treatment, followed by serum hCG less than 15 mIU/mL after six weeks of treatment. In the multiple-dose treatment group, a positive response was defined as a decrease in hCG levels of 15% in 48 hours or after four doses of MTX were given, or serum hCG less than 15 mIU/mL after six weeks of treatment (19 , 20 ). At 12 months after treatment the patients had follow up visits. We telephoned the study participants and questioned them about the outcome of the next pregnancy and of the use of pregnancy prevention methods during the two months following medical treatment.

#### Statistical analysis

Our statistical power calculation showed that 35 patients were needed in each group for 80% power, with side effects of MTX of 20% and an alpha of 5%. Results are presented as mean ± SD or percentile. Statistical analysis was conducted using a Mann-Whitney test, student’s t test for quantified data, and Chi-square for qualitative data. P<0.05 was considered significant. Data analysis was carried out using the Statistical Package for Social Sciences version 19.0 (SPSS, Chicago, IL).

## Results

No statistically significant differences were found between single- and multiple-dose MTX groups in terms of clinical and laboratory characteristics (Table 1). Both single- and multiple-dose MTX groups did not have a statistically significant difference with respect to their initial serum β-hCG concentrations (Table 1), nor was there a significant difference with respect to their serum β-hCG levels seven days after treatment (680 vs. 1100, p=0.326).

Of the 35 patients on the single-dose protocol, treatment was considered successful in 29 (82.9%). Of the patients on the multiple-dose protocol, 31 (88.6%) responded positively to treatment. The difference between success rates in the two groups was not statistically significant (Table 2).

Complications were seen in 8 patients, 2 in the single-dose (5.7%) and 6 in the multiple-dose group (17.2%; p=0.28). The most frequent complaint was hair loss which was observed in 2 (5.7%) patients from the single-dose and 5 (14.3%) from the multidose group. Stomatitis was seen in 1 (2.9%) patient in the multi-dose group. Of the 70 total patients from both groups, 45 had contraception and 25 became pregnant. A total of 9 patients out of 14 in the single-dose group and 3 in the multi-dose regimen became pregnant. Infertility was seen in 4 (33.3%) single-dose patients and 8 (66.7%) multi-dose patients. There was one abortion in the single-dose group (4%; Table 3).

**Table 1:** Comparison of clinical and laboratory characteristics between single-dose and multi-dose treatments with methotrexate (MTX)


	Single-dose (n=35)	Multi-dose (n=35)	P value

**Age (Years)**	28.2 ± 4.1	30 ± 5.8	0.140^a^
**Gestational age (Weeks)**	7.3 ± 1.7	7.2 ± 2.06	0.849^a^
** β-hCG at initiation of treatment (mIU/mL)**	910	1640	0.288^c^
**Endometrial thickness (mm)**	7.3 ± 5.5	8.7 ± 4.1	0.253^a^
**Size of mass on sonography (cm)**	3.2 ± 2.8	2.9 ± 1.9	0.575^a^
**Size of mass on laparoscopic surgery (cm)**	3.2 ± 2.8	2.9 ± 1.9	0.575^a^
**Time that β-hCG level reached <10 (mIU/mL) (Weeks)**	3.3 ± 1.4	3.7 ± 1.3	0.195^a^
**Symptoms on admission:**			0.174^b^
** Vaginal bleeding**	16 (45.7%)	19 (54.3%)	
**Abdominal pain**	6 (17.1%)	6 (17.2%)	
**Amenorrhea**	1 (2.9%)	2 (5.7%)	
** Vaginal bleeding + abdominal pain**	12 (34.3%)	8 (22.8%)	


Data are expressed as mean ± standard deviation, or number of patients with percentages in parentheses. a; Student’s t test, b; Chi-square test and c; Mann-Whitney test.

**Table 2:** Treatment success as defined


	Single-dose (n=35)	Multi-dose (n=35)	P value

**Success rate **	29 (82.9%)	31 (88.6%)	0.587^a^
**Subjects with ruptured EP and selected for surgery**	3 (8.6%)	3 (8.6%)	
**Subjects selected for repeating the treatment**	3 (8.6%)	1 (2.9%)	


a; Chi-square test.

**Table 3:** Fertility outcome after treatment for ectopic pregnancy


	Single-dose (n=14)	Multi-dose (n=11)	P value

**Clinical pregnancy**	9 (75%)	3 (25%)	0.137^a^
**Infertility**	4 (33.3%)	8 (66.7%)	
**Abortion**	1 (4%)	0 (0%)	


a; Chi-square test.

## Discussion

MTX is a folinic acid antagonist that blocks DNA, and to some extent RNA, synthesis, and cell division. As a result, tissues that have a rapid cell turn over, such as trophoblasts, are most sensitive to treatment with MTX (21 ). Although MTX is used in as treatment for EP, its success rate varies in different studies and can increase up to 94% (12 ). van Mello et al. (22 ) have shown that MTX multi-dose intramuscular regimens can be recommended for women with unruptured tubal EP, no signs of active bleeding who present with serum β-hCG concentrations <3000 IU/L. However this regimen is more costly than laparoscopic surgery.

In our study, the success rates of treatment with single-dose MTX was 82.9% and with multi-dose treatment it was 88.6%. Alleyassin et al. (23 ) have shown an 88.9% success rate for single-dose and 92.6% success rate for multi-dose treatment. In a meta-analysis published by Barnhart et al. (12 ), the success rates for single-dose was 88.1% and 92.7% for multi-dose. These researchers noted that none of the trials reviewed were controlled or blinded. In their opinion, a randomized blinded clinical trial would be the most efficient method to reduce potential confounders and bias in comparing the success rate of the two methods.

In one study, multi-dose treatment was reported to be more successful (93% vs. 88%); on the other hand, it was noted that in a single-dose treatment there was no need for folate, a lesser need for monitoring, less side effects (28% vs. 48%), MTX treatment was as effective as laparoscopy, and it was inexpensive (19 ). Soliman et al. (24 ) have reported their success rate as 86.7% and Srivichai et al. (25 ) reported a success rate of 90% for single-dose MTX treatment. They pointed out that in patients with high β-hCG levels prior to treatment and large adnexal mass images on sonography, either multidose MTX or surgical treatment would be preferred. Rozenberg et al. (26 ) found that the success rate of medical treatment was 77.1% and the efficacy of MTX was not improved by the addition of mifepristone.

Potter et al. (27 ) had an 85% overall success rate with single-dose MTX treatment. A higher β-hCG level was associated with a lower success rate of treatment; in addition, they observed that visualization of a yolk sac was a risk factor for treatment failure.

There is evidence that the multi-dose protocol is more successful than the single-dose protocol (12 ), even though it requires more office visits and blood draws. Although the single-dose protocol is simpler, approximately 20% of patients who are treated with this method require at least one additional dose of MTX (12 , 28 -30 ). Other studies have also demonstrated that MTX is a safe, effective medical treatment for unruptured EP, and that life threatening complications have been rare (31 , 32 ).

In our study the two groups were similar according to both clinical and laboratory characteristics. The incidence of infertility was higher in the multiple-dose treatment group. It does not seem to play an important role in the dissimilarity of the two groups, because there was no statistically significant difference in mean gestational age and mean serum β-hCG at the time of diagnosis (33 ). Bouyer et al. (34 ) have noted that the subsequent intrauterine pregnancy rate in the presence of such factors is higher with MTX than with surgical treatment.

Hajenius et al. have demonstrated that in tubal EP, systemic treatment with MTX is as effective as surgery, and the success rate is similar for tubal patency and a future intrauterine pregnancy (35 ). This conflicts with other studies that have shown such factors (history of infertility and contralateral tubal disease) to be associated with a lower subsequent intrauterine pregnancy rate and higher rate of recurrent EP with conservative surgery (36 ). According to other studies, there is no statistically significant difference between single- and multiple- dose treatment regimens in relation to symptoms which were chief complains of patients (37 - 40 ). In our study, the success rate was 82.9% in the single-dose and 88.6% in the multi-dose treatment group. We failed to find a statistically significant difference between the two groups.

## Conclusion

The results of this study have shown that a single- dose MTX protocol can be as successful as a multi-dose protocol for the treatment of EP. A single-dose regimen is also effective, cost-effective, and associated with a better fertility outcome than multi-dose treatment. We believe that further research is warranted before a final decision is reached regarding which medical treatment is best as the initial step in treatment for an unruptured ectopic tubal pregnancy.
